# Surgical Management and Outcomes of Homozygous Familial Hypercholesterolemia in Two Cousins: A Rare Case Report

**DOI:** 10.7759/cureus.11692

**Published:** 2020-11-25

**Authors:** Mutaz M Alenizi, Sulaiman Almushir, Ihab Suliman

**Affiliations:** 1 Medicine, King Abdulaziz Medical City, Ministry of National Guard - Health Affairs, Riyadh, SAU; 2 Medicine, College of Medicine, King Saud Bin Abdulaziz University for Health Sciences, Riyadh, SAU; 3 Cardiology, King Abdulaziz Cardiac Center, King Abdulaziz Medical City, Ministry of National Guard - Health Affairs, Riyadh, SAU

**Keywords:** keywords: surgical, management, homozygous, familial, hypercholesterolemia, case report

## Abstract

Homozygous familial hypercholesterolemia (HoFH) is a rare life-threatening condition characterized by high levels of low-density lipoprotein (LDL) cholesterol in the blood, which increases a person’s risk of developing early atherosclerotic cardiovascular disease (ASCVD). In this report, we present two cases of related patients with aortic stenosis and mitral regurgitation as complications of HoFH. We also discuss the surgical interventions they underwent and their outcomes.

The two related patients with HoFH were admitted to our hospital with signs and symptoms of heart failure. Physical examination revealed an ejection systolic murmur over the aortic valve. Echocardiography revealed valvular disease, and coronary angiography revealed coronary artery disease (CAD). They had undergone the Bentall procedure, mitral valve replacement, and coronary artery bypass graft (CABG) surgery. We elaborate on the progressive course of HoFH, the possible complications associated with this condition, treatment options, and prognosis for the disease.

HoFH is very rare and associated with many cardiovascular complications that can be fatal. The medical treatment of HoFH is rarely sufficient to manage the disease, and surgical interventions are eventually required. The outcomes of surgical treatment are generally good and acceptable.

## Introduction

Familial hypercholesterolemia (FH) is an inherited autosomal dominant genetic disorder that is characterized by high cholesterol levels in the blood, specifically low-density lipoprotein (LDL), which increases a person’s risk of developing early atherosclerotic cardiovascular disease (ASCVD). FH compromises lipid metabolism and clearance because of mutations that affect LDL receptor function, and the most common mutation implicated is the LDL receptor gene mutation [1]. There are two forms of FH. The heterozygous form, which is characterized by having one mutated allele, is the most common form with an incidence of one out of 500, whereas the homozygous form, which is characterized by having two mutated alleles, is much rarer with an incidence of approximately one in a million [2].

Since homozygous familial hypercholesterolemia (HoFH) is caused by two mutated alleles, it is known that this form carries a much worse prognosis compared to the heterozygous form. HoFH is a serious condition that poses a constant threat to the lives of affected individuals through the lifelong exposure to elevated LDL cholesterol levels in the blood, which greatly increases the risk of developing ASCVD, including coronary artery disease (CAD), valvular aortic stenosis, and supravalvular aortic stenosis (SVAS), which are associated with substantial calcium clusters and make surgical intervention challenging [3,4].

Patients with untreated HoFH rarely survive past the age of 20 years, which is why it is important to diagnose these patients appropriately as early as possible, implement reliable screening tools, and provide effective medications as well as aggressive surgical interventions to decrease morbidity and mortality associated with this disease [5]. Since HoFH is very rare, and surgical interventions that are used to manage this condition are both complex and challenging, it is important for healthcare practitioners to learn about real-world patient experience and outcomes of such interventions. In this paper, we report two cases of related patients with aortic stenosis and mitral regurgitation resulting from HoFH, who underwent the Bentall aortic root replacement, mitral valve replacement, and coronary artery bypass graft (CABG) surgery.

## Case presentation

Patient 1

Patient 1 was a 39-year-old male non-smoker who had been diagnosed with HoFH at 14 years of age. He had a positive family history of HoFH in his uncle and three of his first-degree cousins. The patient had been initially treated with oral simvastatin (80 mg) once daily. As the drug had gradually failed to maintain the total and the LDL cholesterol levels within the normal range, he had begun to undergo total plasma exchange and subsequent LDL plasma apheresis at 17 years of age. The combination of LDL plasma apheresis and statin therapy had been effective at that time.

At 24 years of age, he had presented with symptoms of heart failure class II according to the New York Heart Association (NYHA) functional classification, namely, angina and shortness of breath, and physical examination had been unremarkable apart from ejection systolic murmur over the second right intercostal space at the right sternal border. Coronary angiography had revealed an ostial right coronary artery (RCA) lesion with approximately 50% blockage and a left anterior descending artery (LAD) lesion with approximately 55% blockage. His echocardiography had revealed valvular aortic stenosis and SVAS with a pressure gradient of 73 mmHg and a mean gradient of 41 mmHg. The aortic annulus had been 1.6 to 1.8 cm, and his ejection fraction had been more than 55%.

The patient had been admitted at 25 years of age and undergone the Bentall procedure for SVAS, and he had tolerated the procedure very well. On postoperative day three, the patient had collapsed and had been intubated. Coronary angiography had shown stenotic ostial LAD and RCA. The patient had then undergone emergency CABG with the left internal mammary artery (LIMA) to LAD and saphenous vein graft (SVG) to RCA and obtuse marginal artery (OM). Three years after that, the patient had undergone mechanical mitral valve replacement and CABG reoperation (LIMA to diagonal and SVG to RCA and left circumflex artery), and he had tolerated the procedure very well.

**Figure 1:**
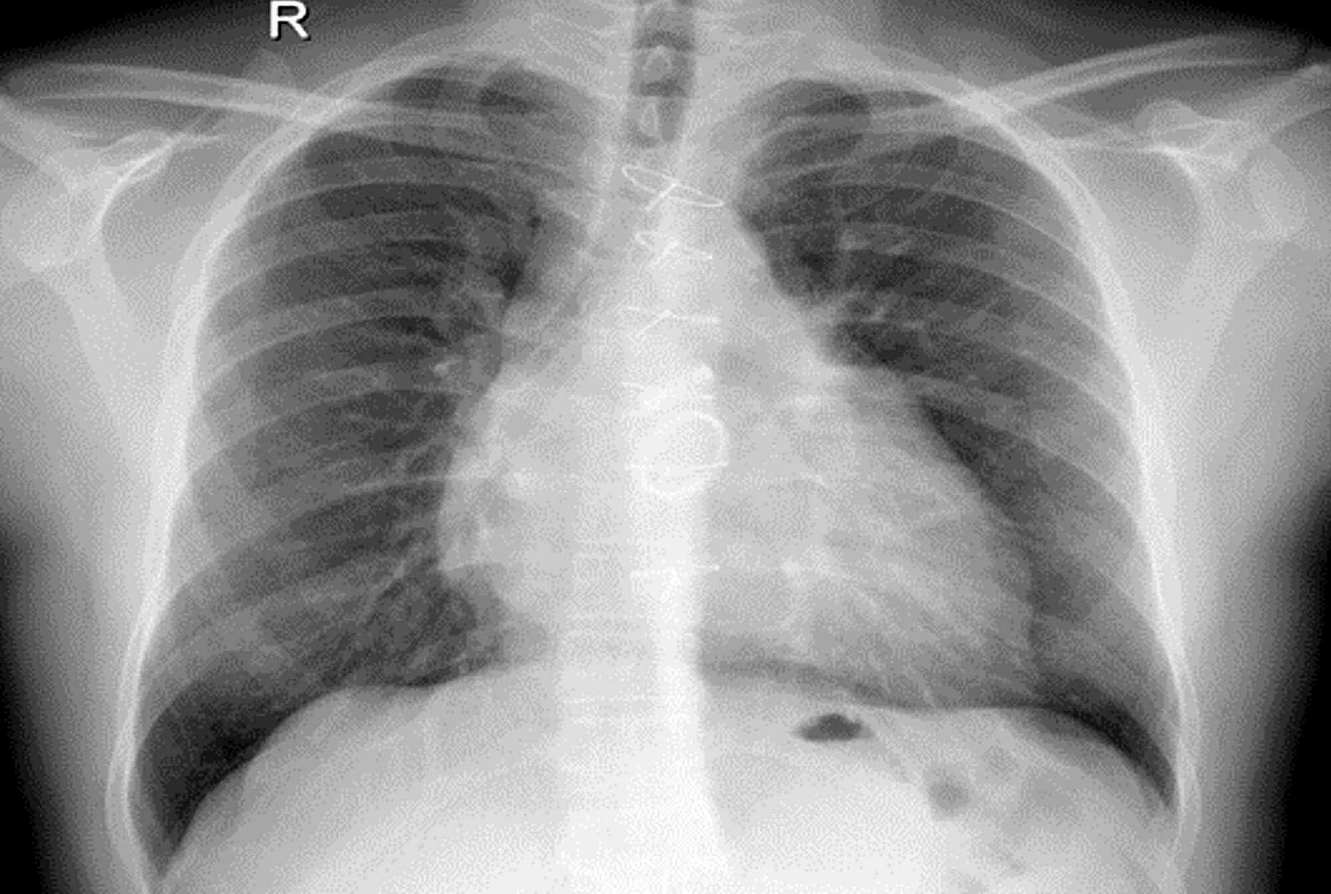
Chest X-ray of patient 1 Status post-sternotomy, cardiac surgery, and valve replacement. Persistent cardiomegaly and minimal pulmonary congestion. Ill-defined opacity in the right lung lower lobe, probably atelectasis/consolidation. Pleural spaces are clear

At the most recent cardiac evaluation (November 18, 2019), the patient was found to be physically active with no significant cardiac symptoms on regular daily activities. He was vitally stable with minimal pulmonary congestion found on his chest X-ray (Figure 1).

**Figure 2:**
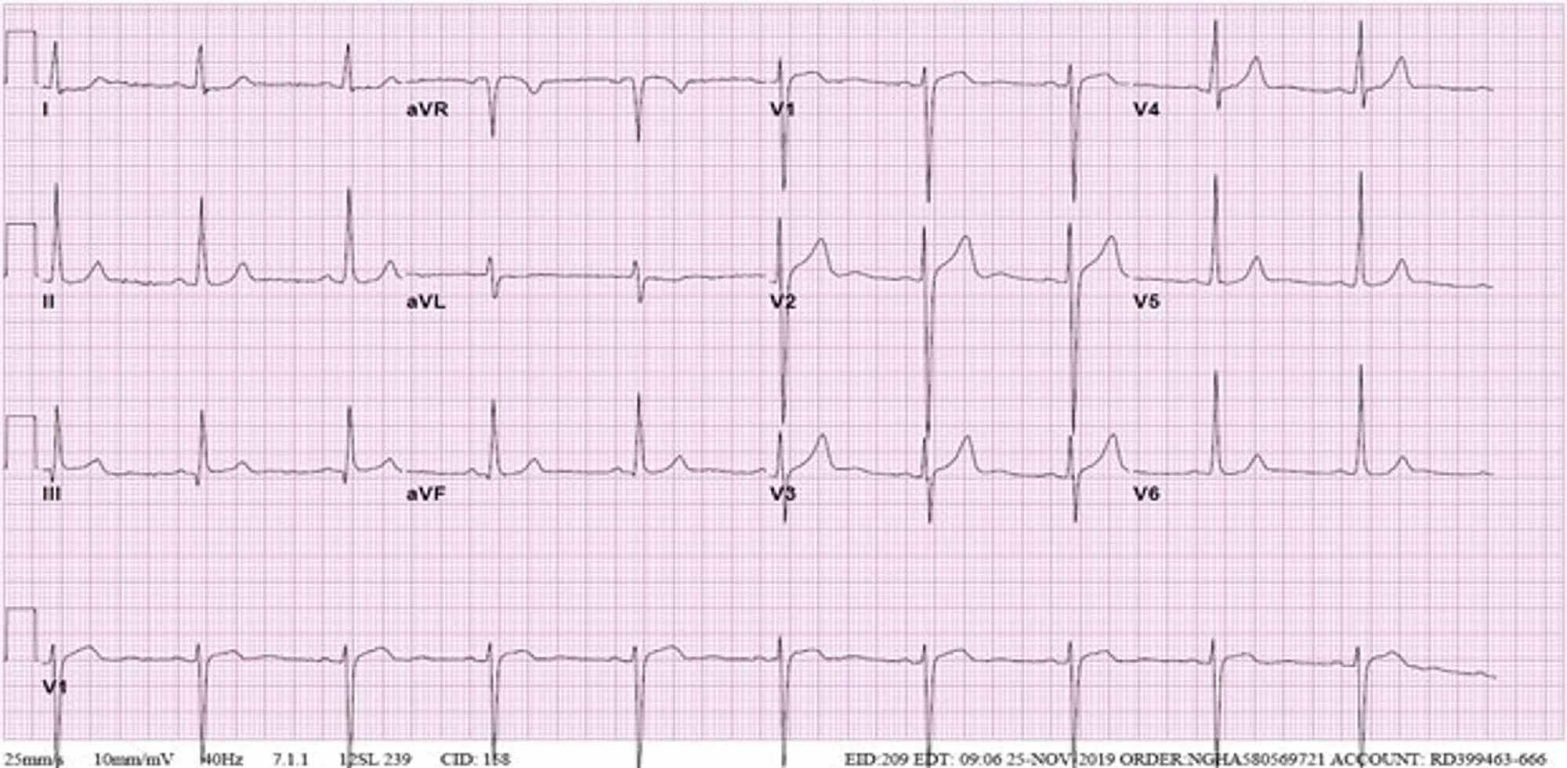
Electrocardiography of patient 1 Normal sinus rhythm. Minimal voltage criteria for left ventricular hypertrophy, maybe a normal variant. Borderline electrocardiography

The patient is currently on regular LDL apheresis through a peripheral line, rosuvastatin, evolocumab, ezetimibe, aspirin, atenolol, and warfarin. The patient’s general condition has been stable with functioning mechanical valves and no rhythm abnormalities on electrocardiogram (ECG) (Figure 2). A timeline of the patient's clinical presentations, diagnostic findings, and treatments over the years is presented in Table 1.

**Table 1:** Timeline of the disease and treatment course for patient 1 LDL: low-density lipoprotein; ECG: electrocardiography; CABG: coronary artery bypass graft

Dates	Clinical presentation	Diagnostic findings	Interventions
April 4, 1994	Angina, dyspnea	Very high LDL cholesterol in serum	Statins
November 9, 1997	Angina, dyspnea	Very high LDL cholesterol in serum	Statins and LDL plasma apheresis
May 10, 2005	Fatigue, palpitation, dyspnea	Coronary angiography revealed blockage and echocardiography revealed valvular and supravalvular aortic stenosis	Bentall procedure for supravalvular aortic stenosis
May 13, 2005	Unconsciousness, cardiac arrest	Coronary angiography showed tight coronary arteries	CABG
July 2, 2008	Angina, dyspnea	Coronary angiography showed tight grafts and echocardiography revealed mitral valve regurgitation	Mechanical mitral valve replacement with redo CABG
November 28, 2019	None	Lipid profile, ECG, Echo, chest X-ray	Statins, LDL apheresis, aspirin, evolocumab, ezetimibe, and warfarin

Patient 2

Patient 2 was a 38-year-old female non-smoker, who was a known case of HoFH that had been diagnosed at 17 years of age. The patient had a positive family history of hypercholesterolemia in her father, sister, and brother. The patient also had a family history of valve replacement in her brother and sister and CABG in her father. Additionally, patient 1 was her first-degree cousin.

At the age of 21, she had presented with symptoms of heart failure (NYHA class II), namely, chest pain and shortness of breath. Physical examination at that time had revealed an ejection systolic murmur over the aortic valve, distended jugular veins, lower limb edema, ascites, confusion, and crackles heard over both lungs. She had undergone a mechanical aortic valve replacement and bioprosthetic mitral valve replacement for aortic stenosis and mitral regurgitation, respectively. During the same admission, the patient had undergone CABG and a Bentall procedure due to significant and symptomatic ischemic changes.

Currently, the patient is on weekly LDL plasma apheresis, atorvastatin, aspirin, evolocumab, ezetimibe, and warfarin. At the most recent cardiac evaluation (January 9, 2020), the patient was stressed according to the Bruce protocol for six minutes, achieving a work level of maximum. The test results were as follows: resting ECG, normal; functional capacity, normal; heart rate response to exercise, appropriate; blood pressure response to exercise, appropriate; chest pain, none; arrhythmias, atrial premature beats triplets, ventricular premature beats; ST changes, depression horizontal; and overall impression, positive stress test typical of ischemia.

For the patient’s grafts, LIMA to LAD was patent but had a small caliber, and the LAD distal to the anastomosis was small. SVG from the proximal RCA to D1 was patent. The graft to the OM was occluded. The aortic root and the ascending aorta were replaced with homografts. There was mild calcification at the descending thoracic aorta. The mitral and aortic replacements were functioning normally. The patient’s general condition is currently stable with functioning valves. A timeline of the patient's clinical presentations, diagnostic findings, and treatments over the years is presented in Table 2.

**Table 2:** Timeline of the disease and treatment course for patient 2 LDL: low-density lipoprotein; ECG: electrocardiography; CABG: coronary artery bypass graft

Dates	Clinical presentation	Diagnostic findings	Interventions
July 7, 1999	Angina, dyspnea	Very high LDL cholesterol in serum	Statins
June 18, 2003	Palpitation, dyspnea	Coronary angiography revealed blockage and echocardiography revealed aortic stenosis and mitral regurgitation	Bentall procedure, mitral valve replacement, and CABG
January 9, 2020	None	Lipid profile, ECG, Echo, chest X-ray	Statins, LDL apheresis, aspirin, evolocumab, ezetimibe, and warfarin

## Discussion

In HoFH, the lifelong exposure to high levels of LDL and total cholesterol in the blood usually lead to CAD and valvular aortic stenosis and SVAS because of lipid infiltration and calcium deposition [3,4]. In our report, patient 1 had undergone two CABG surgeries and two surgeries in which he had both aortic and mitral valves replaced. Patient 2 had undergone CABG once and both aortic and mitral valves had been replaced. The fact that these two patients had been initially controlled with high-dose statin before adding LDL apheresis to their regimen and had finally reached a point where they needed surgical interventions to keep them alive suggests that the progression of their ASCVD was linked to prolonged exposure to the high LDL and total cholesterol levels in the blood rather than their simply having a hereditarily defective cardiovascular system.

The surgical treatment of valvular aortic stenosis and SVAS and CAD in patients with HoFH is very complex and requires excellent facilities and expertise. CAD, for example, maybe difficult to treat because of the calcified aortic wall around the coronary artery ostia, and anastomosing a free graft to the stenotic coronary artery is advisable to protect the myocardium. This might explain why patient 1 collapsed after undergoing the Bentall procedure, and why patient 2, who underwent both the Bentall procedure and CABG at once, did not show the same adverse event that was seen in patient 1.

Although surgical interventions are both invasive and expensive, they are often the only available option for survival in patients with HoFH. In the upcoming years, the prognosis for patients with HoFH might improve with the combination of more potent statins, LDL apheresis, and other newly introduced therapeutic agents. However, as of now, medical treatment does not completely prevent cardiovascular complications, and surgical interventions for CAD and valvular aortic stenosis and SVAS remain the most effective way to prolong survival in these patients.

## Conclusions

HoFH is a very rare condition and it is associated with many cardiovascular complications that can lead to death. The medical treatment of HoFH is rarely enough to manage the disease, and surgical interventions are eventually needed to prolong patient survival. The surgical treatment for HoFH is expensive and technically demanding, but it is often the only available option. The outcomes of surgical treatment are generally good and acceptable in many patients, especially if the patients are compliant with their medications and LDL apheresis sessions after the surgical treatment.
